# Counter-ion effect on antistaphylococcal activity and cytotoxicity of selected antimicrobial peptides

**DOI:** 10.1007/s00726-017-2536-9

**Published:** 2018-01-06

**Authors:** Karol Sikora, Maciej Jaśkiewicz, Damian Neubauer, Marta Bauer, Sylwia Bartoszewska, Wioletta Barańska-Rybak, Wojciech Kamysz

**Affiliations:** 1 Department of Inorganic Chemistry, Faculty of Pharmacy, Medical University of Gdańsk, Al. Gen. J. Hallera 107, 80-416 Gdańsk, Poland; 2 Chair and Clinic of Dermatology, Venereology and Allergology, Medical University of Gdańsk, Gdańsk, Poland

**Keywords:** Counter-ion, Antistaphylococcal activity, Antimicrobial peptides, CAMEL, Citropin 1.1, LL-37, Pexiganan, Temporin A

## Abstract

In view of an appreciable increase in resistance of *Staphylococcus aureus* to the conventional antibiotics, it is desired to develop new effective drugs. Antimicrobial peptides (AMPs) seem to be attractive candidates. In general, AMPs samples used for in vitro studies consist of a peptide, counter-ion, and water. The presence of the counter-ion could be significant as it affects peptide secondary structure and biological activity. The purpose of this study was to estimate the impact of counter-ion on antistaphylococcal activity of selected AMPs (CAMEL, citropin 1.1, LL-37, pexiganan, temporin A). To do this, three kinds of salts were prepared, namely, acetates, hydrochlorides, and trifluoroacetates. In addition, the hemolytic activity against human red blood cells (hRBCs) and cytotoxicity (HaCaT) were determined. The results indicate that there is a substantial difference between different salts, but the pattern is not consistent for the peptides. In general, the antistaphylococcal activity decreased in the order: CAMEL > temporin A > pexiganan > citropin 1.1 ≫ LL-37. The highest selectivity indexes were determined for CAMEL hydrochloride, pexiganan acetate, and temporin A trifluoroacetate. This study shows how important is to take into account the kind of counter-ions when designing novel peptide-based antimicrobials.

## Introduction

Nowadays, the solid-phase synthesis is a popular method in organic chemistry, especially in peptide synthesis (SPPS). Since 1963, when solid-phase method had been introduced by Merrifield, a lot of effort has been expended on SPPS development. Nonetheless, the main features of the method remained unchanged. In general, amino acid derivatives are subsequently attached to an elongated peptide chain and the product is released from the resin with a strong acid. The most popular is Fmoc/*tert*-butyl chemistry where trifluoroacetic acid (TFA) is used for cleavage and final deprotection. Moreover, it is used in RP-HPLC as an additive to mobile phase, e.g., for ion-pairing of basic side chains and *N*-terminus amino group (Chandrudu et al. 2013; Mäde et al. 2014). In effect, synthetic peptides purified by RP-HPLC are obtained as trifluoroacetate salts. Moreover, an excess of the trifluoroacetate ion (together with those anions that are directly bound to positively charged groups) in peptides’ lyophilizate may occur. Importantly, TFA anions are able to affect both the biological and physico-chemical properties of peptides, and for this reason, it is important to consider the counter-ions in peptide studies, including in vivo experiments. To date, several reports regarding the TFA toxicity against cells, e.g., suppression of proliferation of osteoblasts, were reported (Cornish et al. 1999). In fact, counter-ions may also affect the secondary structure of peptides and proteins (Gaussier et al. 2002). The presence of different counter-ions can affect hydrogen-bonding network and alter its structure (Blondelle et al. 1995) (Cinelli et al. 2001). Characterization of synthetic peptides, including conformational analysis, is routinely performed using IR and CD spectroscopy. However, a strong IR band at around 1670 cm^−1^ assigned to TFA which overlaps the amide I band of peptides (1600–1700 cm^−1^) may complicate analysis (Gaussier et al. 2002). Several methods of peptide TFA^−^ counter-ion exchange are described in the literature. A popular one utilizes lyophilization from aqueous solutions of a strong acid, e.g., hydrochloric. As a matter of fact, this method is limited to those anions of acids that are stronger than TFA (with lower pK_a_ values). Other counter-ion exchange methods are based on RP-HPLC, anion-exchange resins, and dialysis through membranes (Roux et al. 2008). Despite the documented influence of counter-ions on the structure and biological activity of molecules, studies on antimicrobial peptides (AMPs) in this area are rather sparse. Nevertheless, the counter-ion effect cannot be ignored, especially because AMPs are positively charged and can interact with anions such as trifluoroacetates. Antimicrobial peptides (AMPs) are the group of compounds that seem to be an alternative to the conventional antibiotics. These evolutionally conserved molecules play a vital role in the innate immune systems of almost all organisms (Mansour et al. 2014). They are targeting a broad spectrum of organisms including bacteria, fungi, protozoa, and viruses, and are able to trigger and coordinate multiple components of innate immunity. For this reason, much attention has been paid to the design of novel, synthetic AMPs (de novo design) with improved properties. A substantial number of AMPs exhibit the antistaphylococcal activity that makes them an object of intense research (Dawgul et al. 2016; Mohamed et al. 2016). Since *Staphylococcus aureus* is still one of the leading pathogens associated with nosocomial and wound infections, it is desired to develop new effective therapies. The aim of this study was to investigate the effect of counter-ion type on antistaphylococcal activity and cytotoxicity of selected AMPs. Peptides used in this study were, CAMEL (Andreu et al. 1992), citropin 1.1 (Wegener et al. 1999), LL-37 (Larrick et al. 1995), pexiganan (Ge et al. 1999), and temporin A (Simmaco et al. 1996). For estimation of the influence of different counter-ions on antistaphylococcal activity, the trifluoroacetate, acetate, and chloride salts were prepared.

## Methods

### Peptide synthesis, purification, and analysis

The tested peptides (cf. Table 1) were synthesized manually by solid-phase method using Fmoc chemistry on the Rink amide or Wang resin (Fields and Noble 1990). All reactions were run using a CEM microwave synthesizer (Liberty Blue) to provide an enhanced efficiency as compared to that obtained by the conventional methodology (Rizzolo et al. 2011). Coupling reaction was carried out by activation with DIC (*N,N’*-diisopropylcarbodiimide) in DMF (*N,N*-dimethylformamide). OxymaPure was applied to suppress racemization instead of HOBt owing to superior coupling efficiencies (Subirós-Funosas et al. 2009). Single deprotection step was accomplished in a 20% piperidine solution in DMF. Deprotection was performed at 75 °C using 30 W for 3 min, whereas the coupling steps were performed at 75 °C using 30 W for 5 min. All reagents were used in a fourfold excess based on the resin. A mixture of TFA, TIS (triisopropylsilane), phenol, and water (92.5:2.5:2.5:2.5, v/v) was used to cleave a peptide from the resin. This reaction was accomplished for 90 min under stirring. The crude peptide was lyophilized and subsequently purified by RP-HPLC. Purifications were carried out on a Phenomenex Gemini-NX C18 column (21.20 × 100 mm, 5.0 µm particle size, and 110 Å pore size). UV detection at 214 nm was used, and the crude peptides were eluted with a linear 10–70% acetonitrile gradient in deionized water over 90 min at room temperature. The mobile phase flow rate was 10.0 mL/min. Acetonitrile and water, both containing 0.1% of TFA, were used as a mobile phase. The purity and identity of the peptide was confirmed by the LC–MS analysis. RP-HPLC system was used—Waters Alliance e2695 system with Waters 2998 PDA and Acquity QDA detectors (software—Empower^®^3). All analyses were carried out on a Waters XBridge™ Shield RP-18 column (4.6 × 150 mm, 3.5 µm particle size, 130 Å pore size). Samples (10 µL) were analyzed with a linear 10–90% acetonitrile gradient in deionized water over 15 min at 25.0 ± 0.1 °C. The mobile phase flow rate was 0.5 mL/min. Both eluents contained 0.1% (v/v) of formic acid. Mass analysis and UV detection at 214 nm were used. Pure fractions (> 95%, by HPLC analysis) were collected and lyophilized.

**Table 1:** Peptides used in this study

Peptide	Sequence	Net charge	Average mass (Da)	MS analysis		
				*z* ^a^	*m* */* *z* ^b^	*m* */z* ^c^
CAMEL	KWKLFKKIGAVLKVL-NH_2_	+6	1771.31	2	886.17	886.85
				3	591.12	591.27
				4	444.59	444.89
Citropin 1.1	GLFDVIKKVASVIGGL-NH_2_	+2	1614.99	2	809.50	808.41
				3	539.34	539.37
LL-37	LLGDFFRKSKEKIGKEFKRIVQRIKDFLRNLVPRTES	+6	4493.33	4	1124.34	1124.31
				5	899.67	899.68
				6	749.90	750.08
				7	642.91	643.19
				8	562.67	562.78
Pexiganan	GIGKFLKKAKKFGKAFVKILKK-NH_2_	+10	2477.21	2	1239.61	1239.39
				3	826.75	826.71
				4	620.31	620.34
				5	496.45	496.52
				6	413.88	414.11
Temporin A	FLPLIGRVLSGIL-NH_2_	+2	1396.78	2	699.40	699.33

*a* Ion charge, *b* calculated mass-to-charge ratio, *c* measured mass-to-charge ratio

### Ion chromatography and counter-ion exchange

Initially, all peptides were obtained as TFA salts. Counter-ions were then exchanged to biocompatible ones: acetates (AcO^−^) and hydrochlorides (Cl^−^). Exchange to AcO^−^ was accomplished in two steps. First, TFA anions were removed with a carbonate ion-exchange resin (Agilent, VariPure columns). Subsequently, the diluted acetic acid was added and the samples were lyophilized. The exchange to Cl^−^ was performed with commonly used method which utilizes the lyophilization from 0.1 M HCl solution (Andrushchenko et al. 2007) or using a HCl-saturated acetonitrile as reported elsewhere (Sikora et al. 2017). With water solutions, exchange was repeated four times and the final products were lyophilized from deionized water to remove an excess of the acid. With saturated acetonitrile, the exchange was repeated twice. Determination of counter-ions and their quantification was done using ion chromatography (Dionex ICS-5000+, Thermo-Scientific). The method was validated for the analysis of TFA^−^, AcO^−^, and Cl^−^ according to the ICH guidelines Q2 (R1) (ICH 2005). The analyses were performed with isocratic elution (4.5 mM Na_2_CO_3_ and 1.4 mM NaHCO_3_ in water), a flow rate of 1.2 mL/min, and the injection volume of 20 µL. All the tested samples were dissolved in water to obtain a concentration of 0.5 mg/mL. Ions were detected by suppressed conductivity with ASRS 300—anion self-regenerating suppressor and the suppressor current of 31 mA. Column characteristics—Dionex IonPac AS22, dimensions 4.0 × 250 mm. Column compartment temperature was set at 30 ± 0.1 °C and conductivity detector temperature was 35 ± 0.1 °C.

### Peptide content

The peptide content was determined spectrophotometrically and the absorbance was measured at 214 nm (Multiskan™ GO Microplate Spectrophotometer, Thermo Scientific). For this purpose, different concentrations of the peptide salts were applied, namely 0.025, 0.050, 0.075, and 0.100 mg/mL to provide evidence that the measurements were accomplished within the linearity range. All samples were dissolved in deionized water and the measurements were conducted in a quartz cuvette with a 10 mm path length. The calculations were done as presented below (Eq. 1):

1 $${\text{Milligram of peptide per milliliter }} = \, \left( {A_{\lambda } \times {\text{ DF }} \times {\text{ MW}}} \right)/e$$

where A_214_—absorbtion at 214 nm [AU], DF—dilution factor, MW—peptide molecular weight [mg × mmole^−1^], and e—molar extinction coefficient at 214 nm [cm^−1^ × M^−1^].

### Organisms and microbiological assay

Microbiological assays were performed using reference and clinical strains of *Staphylococcus aureus*. Minimal inhibitory concentrations (MIC) were determined by broth microdilution method on 96-well polystyrene plates according to Clinical and Laboratory Standards Institute (CLSI) recommendations [Clinical and Laboratory Standards Institute (CLSI) 2012]. For this purpose, the initial inoculums of 0.5 × 10^5^ CFU/mL of bacteria were exposed to a range of concentrations of the peptides (0.5–256 µg/mL). All plates were incubated for 18 h at 37 °C. MIC was taken as the lowest concentration of the compound at which the growth of bacteria was not observed. Reference strains of *S. aureus*: ATCC 6538, ATCC 6538/P, ATCC 9144, ATCC 12598, and ATCC 25923 were obtained from the Polish Collection of Microorganisms (PCM, Polish Academy of Sciences, Wrocław), whereas 24 clinical strains were isolated from the patients of the Clinic of Dermatology, Venereology and Allergology (Medical University of Gdańsk). Nine strains were characterized as methicillin-resistant (MRSA). Preliminary identification and detection was conducted on the ChromID MRSA/ChromID *S. aureus* biplate (bioMérieux) for the simultaneous detection of *S. aureus* and methicillin-resistant *S. aureus* (MRSA). All experiments were conducted at least in triplicate.

### Hemolysis assay

The hemolysis assay was conducted using a procedure described in the literature (Avrahami and Shai 2004). Fresh human red blood cells (RBCs) with EDTA as anticoagulant were rinsed three times with phosphate-buffer saline (PBS) by centrifugation at 800 × g for 10 min and resuspended in PBS. Serial dilution of peptides (1–512 µg/mL) was conducted in PBS on 96-well plates. Then, the stock solution of RBCs was added to reach a final volume of 100 µL with 4% concentration of erythrocytes (v/v). The control wells for 0% hemolysis and 100% hemolysis consisted of RBCs suspended in PBS and 1% of Triton X–100, respectively. Subsequently, the plates were incubated for 60 min at 37 °C and then centrifuged at 800 × g for 10 min at 4 °C (Sorvall ST 16R Centrifuge, Thermo Scientific). After centrifugation, the supernatant was carefully resuspended to new microtiter plates and the release of hemoglobin was monitored by measurement of absorbance at 540 nm (Multiskan™ GO Microplate Spectrophotometer, Thermo Scientific). All experiments were conducted in triplicate.

### MTT assay

To assess the cytotoxicity of the compounds (IC50), the classic MTT assay on 96-well plates was performed for human keratinocytes (HaCaT) obtained from the ATCC. The assay utilizes a colorimetric determination of cell metabolic activity. The color intensity reflects the number of live cells and can be measured spectrophotometrically. Cell line was cultured in DMEM supplemented with 10% FBS (v/v), 100 units/mL of penicillin, 100 μg/mL of streptomycin, and 2 mM l-glutamine, and were kept at 37 °C in a humidified 5% CO_2_ incubator. Briefly, a day after plating of 500 cells per well, different concentrations of the tested compounds were applied (0.5–500 μg/mL). DMSO was added to the control cells at a final concentration of 1.0% (v/v), which was related to the maximal concentration of the solvent compounds used in the experiment. After 24 h of incubation at 37 °C (humidified 5% CO_2_ incubator) with the specified compounds, a medium containing 1 mg/mL of MTT was added to wells to reach a final concentration of 0.5 mg/mL. Subsequently, the plates were incubated at 37 °C for 4 h. Then, the medium was aspirated, and the formazan product was solubilized with DMSO. The absorbance at 630 nm (background absorbance) was subtracted from that at 570 nm for each well (Epoch, BioTek Instruments, USA). Six replicates were conducted for each concentration. All experiments were repeated at least twice and the resulting IC50 values were calculated with a GraFit 7 software.

## Results and discussion

### Peptides synthesis

Information about peptides sequence, net charge, average mass, and the results of the MS analysis are presented in Table 1.

### Counter-ion exchange and determination of counter-ion content

Initially, all peptides were obtained as trifluoroacetate salts and the amount of counter-ions was determined by IC. The level of TFA^−^ ranged between 150 and 320 µg per 1 mg of peptide sample. Moreover, no other anions were detected. Replacement of trifluoroacetates by chlorides was achieved and the level of Cl^−^ anions was above 97 mol% for all peptides. After conversion to chlorides, small amounts of TFA^−^ were found, but further lyophilization from hydrochloric acid did not result in its total elimination. Consistency of all peptides after the exchange was proved by LC–MS analysis. Nonetheless, in case of LL-37, the conversion to chlorides resulted in a lower stability of the peptide. At the beginning, a 0.1 M hydrochloric acid was used. To overcome this problem, we applied an alternative way using HCl-saturated acetonitrile as described elsewhere (Sikora et al. 2017). This modified procedure provided satisfactory conversion to chlorides without peptide degradation. Nevertheless, the stability of the LL-37 chloride turned out to be lower than that of trifluoroacetate and acetate salts, and a slow hydrolysis of the peptide in both the aqueous solution and lyophilizate occurred. This finding suggests that LL-37 hydrochloride inadequate for prolonged storage. In the case of the majority of acetate salts, the level of other anions (TFA^−^ and Cl^−^) was below the limit of detection (LOD) or the limit of quantification (LOQ). Only in samples of temporin A and pexiganan, traces of chlorides were detected. Probably, the chloride as an impurity of the VariPure columns might be bound to the peptides during TFA removal. Results of IC analysis are summarized in Table 2.

**Table 2:** Counter-ions content in peptides (µg/mg and %mol)

Peptide	Desired counter-ion	TFA^−^	AcO^−^	Cl^−^
CAMEL	TFA^−^	272.32/100%	< LOD	< LOQ
	AcO^−^	< LOQ	146.62/98%	3.22/2%
	Cl^−^	6.67/2%	< LOD	98.15/98%
Citropin 1.1	TFA^−^	186.32/100%	< LOQ	< LOQ
	AcO^−^	< LOQ	57.76/100%	< LOQ
	Cl^−^	6.42/3%	< LOD	69.41/97%
LL-37	TFA^−^	155.82/100%	< LOD	< LOD
	AcO^−^	< LOQ	157.52/100%	< LOQ
	Cl^−^	2.30/2%	< LOQ	177.56/98%
Pexiganan	TFA^−^	317.94/100%	< LOD	< LOQ
	AcO^−^	< LOQ	282.43/98%	2.52/2%
	Cl^−^	5.06/1%	< LOD	114.09/99%
Temporin A	TFA^−^	162.54/100%	< LOD	< LOQ
	AcO^−^	< LOQ	63.8/94%	2.47/6%
	Cl^−^	4.73/3%	< LOD	49.28/97%

### Peptide content

The content of the peptides was determined by measurement of absorbance at 214 nm. Their molar extinction coefficients were calculated using appropriate coefficients of peptide building blocks (Kuipers and Gruppen 2007). The results of calculations are summarized in Table 3.

**Table 3:** Molar extinction coefficients of the peptides

Peptide	Molar extinction coefficient at 214 nm [cm^−1^ × M^−1^]
CAMEL	47,653
Citropin 1.1	19,623
LL-37	55,919
Pexiganan	35,702
Temporin A	19,442

The content of the peptides in lyophilizates was calculated for different dilutions within linearity range (0.025–0.1 mg/mL). In Table 4, mean values of the calculated mass fraction, relative standard deviations and relative (percentage) contents are presented. Relative concentration was calculated based on the TFA^−^ salt assumed as 100%. Relative concentration of 2 other salts was calculated on this basis. Moreover, the influence of counter-ions on absorbance was evaluated. Despite the fact that trifluoroacetate has the highest molar extinction coefficient, the absorbance, even at a concentration of 0.1 mg/mL, was insignificant (0.016 AU).

**Table 4:** Peptide content and relative peptide content in peptides’ salts

Peptide	Counter-ion	Mass fraction (mg/mg)	RSD (%)	Relative content
CAMEL	TFA^−^	0.662	1.3	100%
	AcO^−^	0.705	3.1	106.5%
	Cl^−^	0.624	1.2	94.2%
Citropin 1.1	TFA^−^	0.703	3.0	100%
	AcO^−^	0.774	0.3	110.1%
	Cl^−^	0.798	1.5	113.5%
LL-37	TFA^−^	0.719	3.8	100%
	AcO^−^	0.791	2.7	110.1%
	Cl^−^	0.857	1.4	119.2%
Pexiganan	TFA^−^	0.712	1.1	100%
	AcO^−^	0.816	1.9	114.6%
	Cl^−^	0.793	2.6	111.3%
Temporin A	TFA^−^	0.853	0.5	100%
	AcO^−^	0.895	2.7	104.9%
	Cl^−^	0.996	2.7	116.8%

It should be mentioned that molar extinction coefficients are strongly affected by peptide sequence and conformation. Calculation of molar extinction coefficients by adding up extinction coefficients of amino acids and peptide bonds are charged with an error. Consequently, calculation of absolute peptide content cannot be done by measurement of absorbance at 214 nm. Nevertheless, this procedure can be used to compare the content of a particular peptide with different counter-ions. For all peptides, the calculated concentrations ranged from 0.60 to 0.75 mg/mg of lyophilizate. Surprisingly, the high content of the peptide was found for temporin A. Probably, this can be explained in terms of an overestimated value of molar extinction coefficient.

More information could be extracted by comparison of relative concentrations. In relation to TFA^−^ salts, the maximal relative content was found for LL-37 chloride (119.2%). But mostly, the difference between chlorides and acetates in relation to the TFA^−^ form oscillated around 10%. These results indicate that there are no significant differences in peptide content that could affect the activity measured in biological assays.

### Antimicrobial activity

**Fig. 1:**
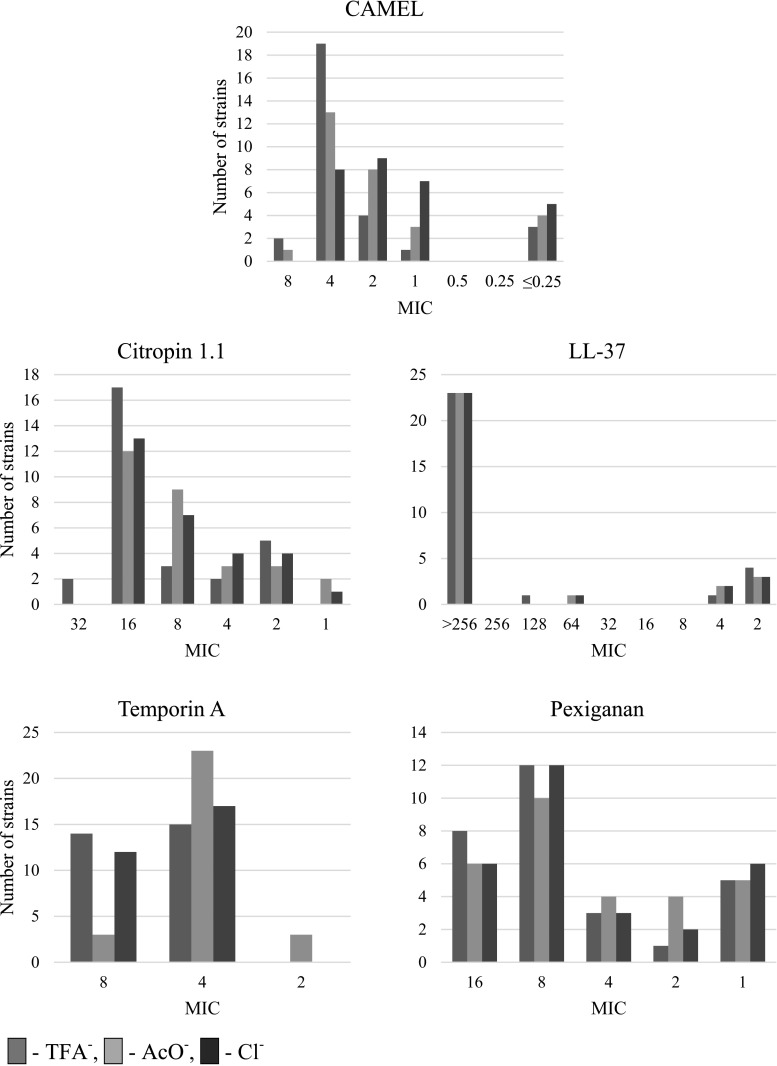
MIC distribution of peptides

All the tested peptides exhibited an antimicrobial activity; although entirely different (Table 3). To asses an overall activity of peptides, the mean MIC values were calculated. In general, the antistaphylococcal activity decreased in the order: CAMEL > temporin A > pexiganan > citropin 1.1 ≫ LL-37. The widest range of MIC values was found for LL-37 (2 to > 512 µg/mL). It seems that in this case, two subpopulations, the resistant (> 512 µg/mL) and sensitive (2–4 µg/mL) strains could have been identified. Moreover, one intermediate strain was recognized, namely, ATCC 6538 (64–128 µg/mL). LL-37-sensitive subpopulation (only clinical isolates) was also susceptible to all AMPs used in this study. The low activity of LL-37 can be explained in terms of interplay of several mechanisms that *S. aureus* bacteria have evolved to resist the innate immune system. One of these are peptidases and proteases that are capable to hydrolyse AMPs; for example, the metalloprotease aureolysin and the serin protease V8 which exhibit high affinity towards LL-37 (Sieprawska-Lupa et al. 2004)(Kraus and Peschel 2008). The MIC ranges for peptides among strains are as follows: CAMEL, ≤ 0.25–8 µg/mL; citropin 1.1, 1–32 µg/mL; pexiganan, 1–16 µg/mL; and for temporin A, 2–8 µg/mL. At first sight, it seems unclear how antistaphylococcal activity among the strains can differ between peptide salts. A better explanation can be provided by calculated mean MIC values (Table 5) and MIC distribution (Fig. 1). In case of all peptides, the lowest antimicrobial activity was found for trifluoroacetate salts. Moreover, MIC distribution for acetates and chlorides differs from that of trifluoroacetates. Importantly, distribution of MIC values for chlorides and acetates is shifted toward lower concentrations. Both peptide salts, the acetates and chlorides, seem to be more potent antimicrobials than trifluoroacetates; but, a decision on the superiority of one salt over the other cannot be taken. Interestingly, this conclusion could have been made owing to the significant number of the strains tested. In other words, the change in activity is strain-dependent, and only experiments carried out on different bacterial isolates can provide convincing results.

**Table 5:** MIC values of the peptides [µg/mL]

Peptide and counter-ion	CAMEL			Citropin 1.1			LL-37			Pexiganan			Temporin A		
	TFA^−^	AcO^−^	Cl^−^	TFA^−^	AcO^−^	Cl^−^	TFA^−^	AcO^−^	Cl^−^	TFA^−^	AcO^−^	Cl^−^	TFA^−^	AcO^−^	Cl^−^
*S. aureus* (29)															
Reference (5)															
ATCC 25923	8	8	2	32	16	16	>	>	>	8	8	8	4	8	4
ATCC 6538	2	≤	≤	16	4	4	128	64	64	4	2	4	4	4	4
ATCC 6538/P	4	1	1	16	8	8	>	>	>	8	4	8	8	4	8
ATCC 9144	8	2	1	32	16	16	>	>	>	8	8	8	8	8	8
ATCC 12598	4	2	1	16	8	8	>	>	>	16	8	8	4	4	4
Clinical (24)															
001N (MRSA)	4	2	2	16	16	16	>	>	>	16	8	8	8	4	8
001S (MRSA)	4	4	2	16	16	16	>	>	>	8	8	8	4	4	4
002N	4	4	≤	16	16	16	>	>	>	8	16	16	4	4	4
002S	4	4	1	16	16	8	>	>	>	16	16	16	4	4	4
004N (MRSA)	4	4	2	16	8	8	>	>	>	8	4	8	4	4	4
005S	≤	≤	≤	2	2	2	2	2	4	1	1	1	4	4	4
013S	≤	≤	≤	2	2	2	2	4	2	1	1	1	4	4	4
015N (MRSA)	4	4	2	16	8	8	>	>	>	16	4	4	8	4	8
017N (MRSA)	4	2	2	8	4	4	>	>	>	4	2	2	8	4	8
017S (MRSA)	2	2	2	4	8	4	>	>	>	4	2	2	8	4	8
024N (MRSA)	≤	≤	≤	2	2	2	2	4	2	1	1	1	4	4	4
030N	4	4	4	16	16	16	>	>	>	16	16	8	4	4	4
033N	4	4	4	16	8	16	>	>	>	16	8	16	4	2	4
039N	4	4	4	16	8	16	>	>	>	8	16	8	4	2	4
043SC (MRSA)	4	4	4	8	8	8	>	>	>	8	4	4	8	4	8
045N	4	4	4	16	16	16	>	>	>	16	8	16	8	4	8
048S	4	4	4	16	16	16	>	>	>	16	16	16	4	4	4
051N (MRSA)	4	2	2	8	8	8	>	>	>	8	8	8	8	4	8
051S	2	2	2	4	4	4	>	>	>	1	1	1	8	4	8
053N	4	4	4	16	16	16	>	>	>	8	8	8	8	4	4
060S	2	1	1	2	1	2	2	2	2	2	1	1	4	2	4
K19N	4	2	1	16	16	16	>	>	>	8	16	16	8	8	8
K46N	4	4	4	16	16	16	>	>	>	8	8	8	8	4	8
K50S	1	1	1	2	1	1	4	2	4	1	2	1	8	4	4
Mean value of MIC	≤ 3.5	≤ 2.8	≤ 2.0	13.0	10.0	10.0	> 411	> 409	> 409	8.4	7.1	7.4	5.9	4.2	5.7

≤ *alone* stands for MIC ≤ 0.25 µg/mL; > *alone* stands for MIC > 256 µg/mL

MIC distribution of peptides against all *S. aureus* strains used in this study is shown in Fig. 1.

### Hemolysis assay

**Fig. 2:**
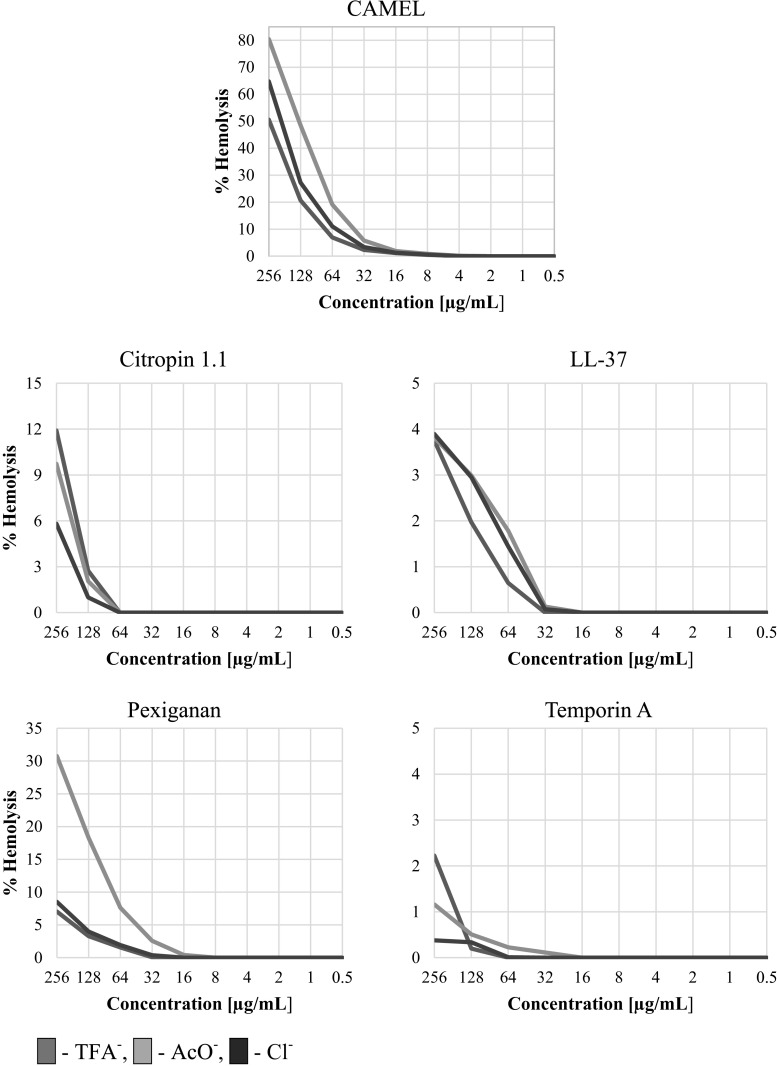
Percentage of hemolysis of erythrocytes vs peptide concentration

Peptides used in this study exhibited different profiles of hemolysis. We can classify AMPs by increasing hemolysis in the order: temporin A < LL-37 < pexiganan (TFA^−^, Cl^−^) < citropin 1.1 < pexiganan (AcO^−^) < CAMEL. In fact, hemolysis of temporin A and LL-37 did not exceed 4% even at their highest concentration of 256 µg/mL. On account of their irrelevant toxicity towards human RBCs, those peptides have been excluded from further analysis of counter-ion effect on hemolysis. The results indicate that peptide trifluoroacetates are not as strongly hemolytic as it had been expected. Our results show that acetate counter-ions can substantially contribute to the hemolysis. For instance, the hemolysis of RBCs by pexiganan acetate (30.75% at 256 µg/mL) is substantially higher than that by both trifluoroacetate (7.04%) and chloride (8.51%). Moreover, similar conclusions can be applied to CAMEL. The chloride salts of the peptides seem to be the most suitable form owing to a noticeable increase in antistaphylococcal activity and moderate changes in hemolysis (Fig. 2).

### Cytotoxicity: MTT assay

All the tested peptides exhibited a relatively high cytotoxicity against HaCaT cell line. The IC50 values ranged between 0.14 and 22.65 µg/mL. Most of the determined IC50 values were lower than the mean MIC values, this being a serious drawback. In the case of CAMEL and citropin 1.1, counter-ion type had a little impact on cytotoxicity. With temporin A, the trifluoroacetate proved to be the least toxic, while the acetate was the most. Interestingly, for three peptides, citropin 1.1, LL-37, and pexiganan, the lowest cytotoxicity was found for the acetate salts. These results are not consistent with the hemolysis pattern. In fact, AMPs acetates (CAMEL, pexiganan) exhibited the highest toxicity against RBCs. The calculated selectivity index confirmed different selectivity of the peptide salts towards *S. aureus* strains (Table 6).

**Table 6:** IC50 values and selectivity indexes of the peptides

Peptide	IC_50_	Mean MIC values	Selectivity index (IC50/MIC)
CAMEL			
TFA^−^	3.32	≤ 3.5	≥ 0.95
AcO^−^	2.72	≤ 2.8	≥ 0.97
Cl^−^	2.49	≤ 2.0	**≥** **1.25**
Citropin 1.1			
TFA^−^	3.02	13.0	0.23
AcO^−^	4.37	10.0	0.44
Cl^−^	4.12	10.0	0.41
LL-37			
TFA^−^	11.75	> 411	< 0.03
AcO^−^	22.65	> 409	< 0.06
Cl^−^	4.26	> 409	< 0.01
Pexiganan			
TFA^−^	1.05	8.4	0.13
AcO^−^	10.60	7.1	**1.49**
Cl^−^	0.14	7.4	0.02
Temporin A			
TFA^−^	7.91	5.9	**1.34**
AcO^−^	0.92	4.2	0.22
Cl^−^	3.43	5.7	0.60

Bold values are important in relation to the remaining results

CAMEL was highly effective against all the tested strains with mean MIC value below 3.5 µg/mL for the three salts. According to the selectivity index, the most convenient would be the chloride. Unfortunately, its antimicrobial activity and toxicity against HaCaT cell line are similar, thus limiting its application. All citropin 1.1 salts exhibited high cytotoxicity in the MTT assay and high activity against *S. aureus* strains, both reference and clinical isolates. However, the selectivity index suggests a limited application of these peptides in the treatment of *S. aureus* infections. As previously stated, LL-37 exhibited low antistaphylococcal activity accompanied by a low hemolytic activity in all salt forms. Nevertheless, other studies on LL-37 (Johansson et al. 1998) provide evidence that the counter-ion may induce changes in peptide helical content and thus its antimicrobial activity against both Gram-positive *Bacillus megaterium Bm11* and Gram-negative *Escherichia coli* D21. Moreover, impact of trifluoroacetate and chloride on the peptide helicity proved to be entirely different. It has been stated that TFA^−^ has greater ability to promote helix formation in LL-37 than does Cl^−^. In comparison to other forms (Cl^−^ and TFA^−^), pexiganan acetate showed the highest selectivity against *S. aureus* with selectivity index of 1.49. Interestingly, a formulation of pexiganan acetate was patented in 2015 by Dipexium Pharmaceuticals company (Nayan Desai 2013). The authors suggested that the acetate form is the most stable even during prolonged storage, and the formulation of this peptide can be applied in the treatment of wound and skin infections often accompanied with presence of *S. aureus*. The MIC and IC50 values seem to confirm assumption that the pexiganan acetate is a promising candidate for further clinical tests. Moreover, this compound exhibited the lowest toxicity in the MTT assay and the hemolysis of RBCs at the mean MIC value was ignorable.

Temporin A showed a relatively high antimicrobial activity against *S. aureus* and almost undetectable hemolysis of RBCs. The calculated selectivity index showed that the greatest ratio of antistaphylococcal activity-to-cytotoxicity was found for TFA salts (1.36), whereas temporin A acetate and chloride revealed no selectivity (0.22 and 0.60, respectively).

## Conclusions

Almost all the AMPs tested in this study exhibited a high activity against reference and clinical strains of *S. aureus.* Moreover, essential differences in antimicrobial, hemolytic activity, and cytotoxicity were found between the tested salts. In general, LL-37 showed weak antistaphylococcal properties, but three sensitive isolates were identified. The greatest antistaphylococcal activity was noticed for CAMEL with a slight superiority of chloride form. On the other hand, this peptide showed the highest hemolytic activity among all the tested peptides and a relatively high cytotoxicity. Nonetheless, pexiganan acetate and temporin A trifluoroacetate exhibited the highest selectivity index among the tested salts. Surprisingly, pexiganan acetate is the one showing the highest hemolytic activity against human RBCs. The results obtained in this study suggest that there is no simple correlation between the type of counter-ion in the peptide and the biological activity. We believe that each case should be considered individually due to peptide-dependent differences between salts. Nevertheless, our findings undoubtedly support the thesis that the kind of the counter-ion is critical for biological properties of antimicrobial peptides.

## Abbreviations

ACN: Acetonitrile
AMPs: Antimicrobial peptides
ATCC: American Type Culture Collection
CD: Circular dichroism
CFU: Colony forming units
CLSI: Clinical and Laboratory Standards Institute
DCM: Dichloromethane
DIC: *N,N′*-diisopropylcarbodiimide
DMEM: Dulbecco’s Modified Eagle’s Medium
DMF: *N,N*-dimethylformamide
DMSO: Dimethyl sulfoxide
EDTA: Ethylenediaminetetraacetic acid
ESI–MS: Electrospray-ionization mass spectrometry
FBS: Fetal bovine serum
Fmoc: 9-Fluorenylmethoxycarbonyl group
HaCaT: Immortalized human keratinocytes
HOBt: 1-Hydroxybenzotriazole
IC: Ion chromatography
IC50: Half maximal inhibitory concentration
IR: Infrared radiation
LOD: Limit of detection
LOQ: Limit of quantification
MIC: Minimal inhibitory concentration
MRSA: Methicillin-resistant *Staphylococcus aureus*
MTT: 3-(4,5-Dimethyl-2-thiazolyl)-2,5-diphenyl-2H-tetrazolium bromide
PBS: Phosphate-buffer saline
PCM: Polish collection of microorganisms
RBCs: Red blood cells
RP-HPLC: Reverse-phase high-performance liquid chromatography
SPPS: Solid-phase peptide synthesis
TFA: Trifluoroacetic acid
TIS: Triisopropylsilane
